# Microwave ablation and synchronous transarterial chemoembolization combined with PD-1 inhibitor in patients with hepatocellular carcinoma following tyrosine kinase inhibitor intolerance

**DOI:** 10.3389/fimmu.2022.1097625

**Published:** 2023-01-10

**Authors:** Qin Shi, Xin Zhou, Zihan Zhang, Wen Zhang, Jingqin Ma, Minjie Yang, Jiaze Yu, Jianjun Luo, Lingxiao Liu, Zhiping Yan

**Affiliations:** ^1^ Department of Interventional Radiology, Zhongshan Hospital, Fudan University, Shanghai, China; ^2^ Department of Interventional Radiology, Shanghai Institution of Medical Imaging, Shanghai, China; ^3^ National Clinical Research Center for Interventional Medicine, Zhongshan Hospital, Fudan University, Shanghai, China

**Keywords:** hepatocellular carcinoma, microwave ablation, transarterial chemoembolization, PD-1 inhibitor, survival

## Abstract

**Purpose:**

To determine the safety and efficacy of microwave ablation (MWA) and synchronous transarterial chemoembolization (TACE) combined with or without PD-1 inhibitor in patients with hepatocellular carcinoma (HCC) following tyrosine kinase inhibitor (TKI) intolerance.

**Materials and methods:**

This study retrospectively enrolled TKI-intolerant HCC patients who underwent MWA-TACE combined with PD-1 inhibitor (MTP) or MWA-TACE (MT) from January 2019 to June 2021. MWA and TACE were performed simultaneously, and PD-1 inhibitor was administered intravenously at a dose of 200 mg once every three weeks after MWA-TACE. Adverse events (AEs) related to treatment were recorded during the follow-up. Progression-free survival (PFS) and overall survival (OS) were compared between the two groups.

**Results:**

A total of 87 patients were included and classified into the MTP group (n =42) and MT group (n=45). Complications related to MWA-TACE in the MTP group were similar to that in the MT group (21.4% vs. 24.4%, *P* = 0.738). Moreover, 35 (83.3%) patients had eighty-four AEs related to PD-1 inhibitor in the MTP group, and 8 (19.0%) patients developed grade 3. Patients who underwent MWA-TACE combined with PD-1 inhibitor had better PFS (median, 10.0 vs. 4.7 months, *P* < 0.001) and OS (median, 17.0 vs. 8.5 months, *P* < 0.001) than those who underwent MWA-TACE alone. Treatment method and Child-Pugh class were independent prognostic factors for survival in the univariate and multivariate analysis.

**Conclusion:**

MWA and synchronous TACE combined with PD-1 inhibitor might be a favorable treatment option in TKI-intolerant HCC patients.

## Introduction

Hepatocellular carcinoma (HCC) is the sixth most common malignant tumor and the third leading cause of cancer death worldwide (1, 2). Despite continuous improvements in diagnostic techniques, a large number of patients are in the progression stage at the time of detection and are not candidates for curative therapies such as liver resection and transplantation (3, 4). Systemic therapies, such as tyrosine kinase inhibitors (TKIs), have been widely used in patients with HCC, especially at advanced stage (5). However, some patients are prone to TKI intolerance due to serious side effects. The optimal treatment remains unknown and controversial for these patients.

Transarterial chemoembolization (TACE) is considered as the mainstay of treatment for unresectable HCC, especially for large or multifocal tumors (6). The theoretical basis for TACE is a synergy between ischemia and deposition of chemotherapeutic drugs several times using appropriate concentrations. Microwave ablation (MWA) is the common thermal ablation treatment for HCC (7, 8). It is based on the principle of continuous realignment of polar water molecules within the ablation zone by generating an oscillating electromagnetic field, which causes friction within the tissue and leads to coagulative necrosis (9). Some studies demonstrated that MWA could rapidly produce larger and hotter ablation zones than radiofrequency ablation (RFA), making it less susceptible to the heat sink effect with a shorter ablation time (10, 11). In recent years, the combination of TACE and thermal ablation has benefited some HCC patients. Yin et al. (12) reported that TACE combined with RFA could improve the efficacy in patients with intermediate-stage HCC. In addition, a retrospective study of 231 HCC patients showed that TACE combined with MWA provided a survival benefit over TACE alone for HCC patients beyond the Milan criteria (13).

As emerging cancer immunotherapy, immune checkpoint inhibitors (ICIs) have attracted more and more attention in patients with HCC. The IMBrave 150 trial demonstrated that atezolizumab combined with bevacizumab had better overall and progression-free survival outcomes than sorafenib in unresectable HCC (14). In the 2022 updated BCLC guideline, the combination of atezolizumab and bevacizumab was recommended as a first-line treatment for advanced HCC (15). Since then, the combination of ICIs has been explored in preclinical and clinical practice. Programmed death 1 (PD-1) and its ligand (PD-L1/PD-L2) inhibitor are the most widely used ICIs. The mechanism is to specifically block the combination of PD-1 and PD-L1/PD-L2, and restores the function of effector T cells to induce tumor cytotoxicity. Huang et al. (16) confirmed that the combination of PD-1 inhibitor and MWA could inhibit the distant tumor growth and construct a systemic anti-tumor immune environment in a multitumor Hepa1-6 murine model. Therefore, the present study aimed to determine the safety and efficacy of MWA and synchronous TACE combined with PD-1 inhibitor in TKI-intolerant HCC patients.

## Materials and methods

### Study design and population

The study was approved by the Ethics Committee of Zhongshan Hospital, Fudan University, and was conducted according to the guidelines of the Declaration of Helsinki. The electronic medical records of TKI-intolerant HCC patients who underwent MWA-TACE combined with PD-1 inhibitor (MTP) or MWA-TACE alone (MT) from January 2019 to June 2021 were reviewed. Diagnosis of HCC was based on non-invasive criteria in accordance with the European Association for the Study of Liver or American Association for the Study of Liver Disease guidelines (5, 17). The multidisciplinary team recommended the treatment strategy of MWA-TACE combined with or without PD-1 inhibitor.

Inclusion criteria for this study were as follows: (a) patients with HCC who were intolerant to TKI such as sorafenib or lenvatinib, (b) Child-Pugh class A or B, (c) Eastern Cooperative Oncology Group (ECOG) performance status of 0-2, (d) patients underwent at least one session of MWA-TACE combined with PD-1 inhibitor in our institution. Patients were excluded from the study if they (a) had incomplete medical records, (b) had serious comorbidities including hepatic encephalopathy, refractory ascites, and esophageal variceal bleeding, (c) had a history of malignant tumors in addition to HCC, (d) had other liver-directed therapy during the study period.

### MWA-TACE procedure

MWA and TACE were performed simultaneously in the study. At first, hepatic angiography was conducted to evaluate the tumor size, location, number, and arterial blood supply. Then, a commercially available water-cooled microwave system (VISON-CHINA MEDICAL DEVICES R&D CENTER, USA) was used to perform MWA. After administration of local anesthesia, one or two antennae were inserted into the tumor *via* ultrasound guidance. The position of the antennae, power output setting and ablation time were determined based on the tumor size, number, and distance from vulnerable structures (gallbladder, diaphragm, or gastrointestinal tract). The ablation procedure was repeated until the hyperechoic region overlapped with the tumor area under ultrasound guidance. The needle track was coagulated to prevent bleeding or tumor seeding. Afterward, arteriography was performed again to evaluate the residual tumor stain and ablative results. Subsequently, superselective catheterization of residual tumor blood supply was performed. The emulsion of chemotherapeutic agents (10-30mg epirubicin or 20-50 mg lobaplatin) and 5-10 mL lipiodol were administered *via* tumor-feeding artery. Finally, embolization was performed with blank microspheres or gelatin sponge particles until arterial flow stasis under X-ray.

### PD-1 inhibitor administration

The PD-1 inhibitors including Camrelizumab, Pembrolizumab and Sintilimab injection were used in this study. The administration was injected intravenously at a dose of 200 mg once every three weeks after MWA-TACE. The interruption and discontinuation of PD-1 inhibitor administration depended on the patient’s status and the severity of drug toxicity.

### Follow-up

The deadline for follow-up was December 31, 2021. All patients underwent regular follow-up at 4-6 weeks after treatment. The contents mainly included detailed medical records, laboratory tests, and dynamic CT or MRI. During follow-up, MWA-TACE and PD-1 inhibitor would be repeated if the residual or recurrent tumor was visible on dynamic CT or MRI. The treatment strategy was discontinued and changed when intolerable toxicity occurred. The subsequent treatment, such as radiotherapy, iodine-125 seed brachytherapy, hepatic artery infusion chemotherapy (HAIC), or best supportive care, was determined by the multidisciplinary team.

### Assessment

Overall survival (OS) and progression-free survival (PFS) were recorded and compared between the MTP group and MT group. OS was defined as the period from the initial treatment to death or the last follow-up. PFS was defined as the period from the initial combination therapy until the time of radiological progression or death.

The safety of all patients who underwent MWA and synchronous TACE was assessed by using the Society of Interventional Radiology classification system (18). A major complication was defined as an event that resulted in substantial morbidity and disability, which increased the level of care, led to hospital admission, or substantially lengthened the hospital stay. All other complications were considered minor. Symptoms of postembolization syndrome (such as abdominal pain, fever without any infection focus, nausea and vomiting) were expected. Therefore, they were not documented separately. AEs related to PD-1 inhibitor were recorded and assessed according to the National Cancer Institute Common Terminology Criteria, version 5.0.

Tumor response was evaluated according to the modified Response Evaluation Criteria in Solid Tumors (mRECIST) criteria (19). The objective response rate (ORR) was defined as the percentage of the sum of complete response (CR) and partial response (PR). The disease control rate (DCR) was expressed as the percentage of the sum of CR, PR, and stable disease (SD). Intrahepatic targeted tumor response only referred to changes in tumors inside the liver after treatment. And overall tumor response evaluated changes in all tumors, including liver and extrahepatic tumors.

### Statistical analysis

Statistical analyses were performed using SPSS version 25.0 and GraphPad Prism version 8.0. Continuous variables were expressed as mean ± standard deviation, and categorical variables were expressed as numbers and percentages. The independent sample *t* test, Person *χ* ^2^, continuity correction and Fisher’s exact test were used to compare the differences between the MTP group and MT group. Survival curves for OS and PFS were performed using the Kaplan-Meier method. Univariate analyses were performed to identify the predictive factors of survival using the log-rank test. Variables with a *P* value less than 0.10 in the univariate analysis were entered into the multivariate analysis using Cox proportional hazard regression model. All analyses were two-tailed. A *P* value less than 0.05 was considered significant.

## Results

### Patient characteristics

**Figure 1:**
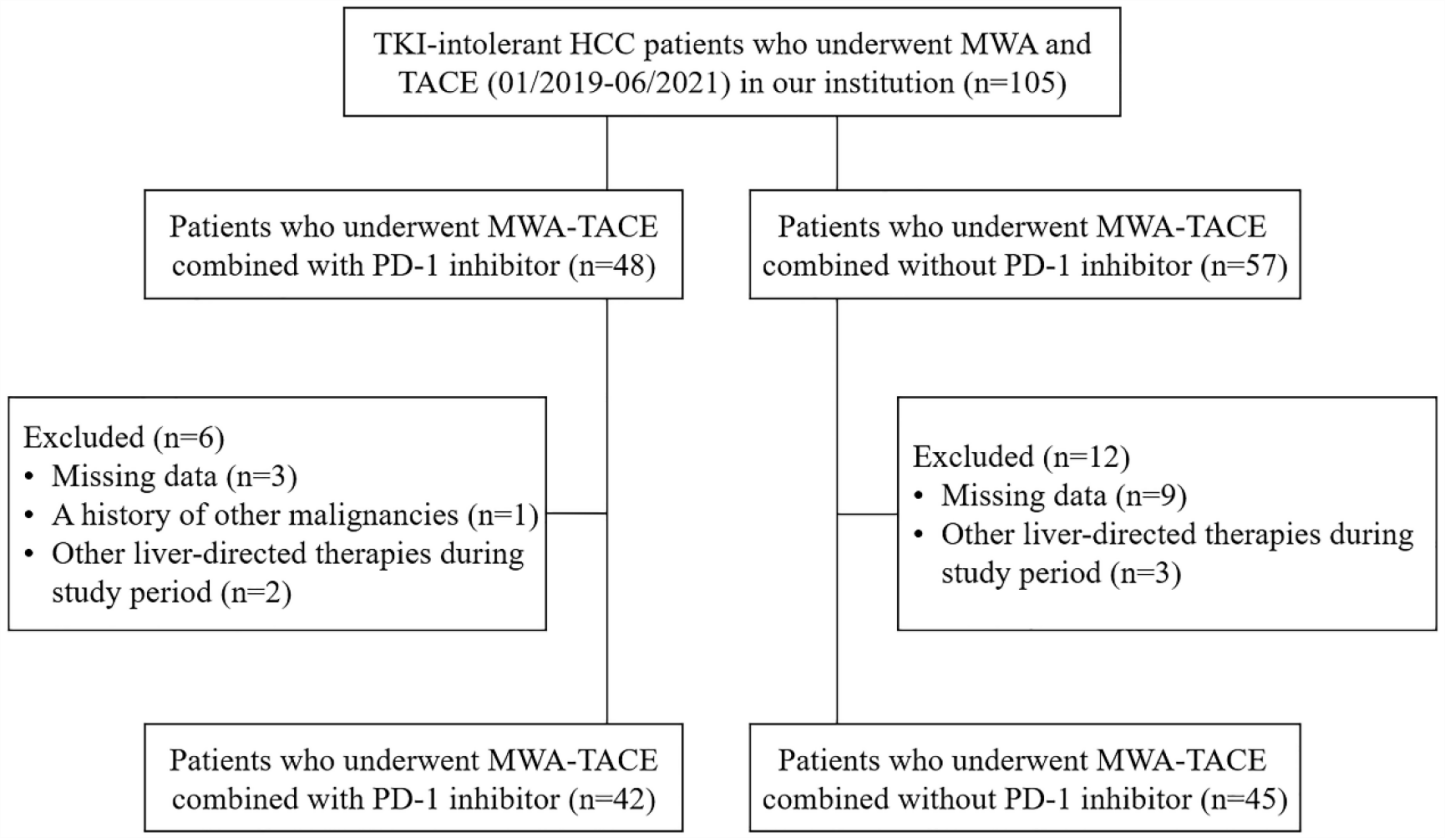
Flow diagram of patient enrollment. HCC, hepatocellular carcinoma; MWA, microwave ablation; TACE, transarterial chemoembolization.

According to the inclusion and exclusion criteria, a total of 87 patients who underwent MWA-TACE combined with PD-1 inhibitor (n=42) or MWA-TACE alone (n=45) were included in this study (**Figure 1**). Detailed baseline characteristics of these patients are shown in **Table 1**. The baseline characteristics were not significantly different between the two groups.

**Table 1:** Baseline Characteristics of all patients.

Variable	MTP group (n=42)	MT group (n=45)	*P* value
Sex			0.601
Male	38 (90.5)	38 (84.4)	
Female	4 (9.5)	7 (15.6)	
Age (y)	55.9 ± 10.6	58.4 ± 11.7	0.313
Etiology			0.318
Hepatitis B infection	33 (78.6)	39 (86.7)	
Other	9 (21.4)	6 (13.3)	
Tumor location			0.894
Unilobar	19 (45.2)	21 (46.7)	
Bilobar	23 (54.8)	24 (53.3)	
Child-Pugh class			0.232
A	36 (85.7)	34 (75.6)	
B	6 (14.3)	11 (24.4)	
ALBI grade			0.695
1	16 (38.1)	19 (42.2)	
2	26 (61.9)	26 (57.8)	
Maximal tumor diameter (cm)	8.8 ± 4.2	8.9 ± 4.2	0.910
No. of tumors			0.629
≤ 3	24 (57.1)	28 (62.2)	
> 3	18 (42.9)	17 (37.8)	
Portal vein tumor thrombosis	26 (61.9)	36 (80.0)	0.062
Hepatic vein invasion	10 (23.8)	12 (26.7)	0.759
Extrahepatic spread	11 (26.2)	9 (20.0)	0.493
Laboratory test			
Total bilirubin level (μmol/L)	13.7 ± 6.2	19.9 ± 24.3	0.104
Albumin level (g/L)	38.3 ± 4.9	38.5 ± 4.4	0.859
ECOG PS			0.172
0	31 (73.8)	27 (60.0)	
1-2	11 (26.2)	18 (40.0)	
BCLC stage			0.318
B	9 (21.4)	6 (13.3)	
C	33 (78.6)	39 (86.7)	
α-fetoprotein level			0.073
< 400 ng/mL	22 (52.4)	15 (33.3)	
≥ 400 ng/mL	20 (47.6)	30 (66.7)	

MTP, microwave ablation and transarterial chemoembolization combined with PD-1 inhibitor; MT, microwave ablation and transarterial chemoembolization; ALBI, albumin-bilirubin; ECOG PS, Eastern Cooperative Oncology Group performance status; BCLC, Barcelona clinical liver cancer.

The mean follow-up duration was 15.2 months (range, 5.8-27.2 months) in the MTP group and 8.7 months (range, 2.0-19.3 months) in the MT group. 34 (81.0%) patients in the MTP group and 30 (66.7%) patients in the MT group underwent subsequent local treatment, which mainly included repeated MWA-TACE, TACE or HAIC alone. In addition, 25 (59.5%) patients in the MTP group died during the observation period, of which 15 from disease progression, 8 from gastrointestinal bleeding, 2 from accidents. And 43 (95.6%) patients in the MT group died during the follow-up period, of which 36 patients from disease progression, 5 from gastrointestinal bleeding, 2 from heart attack. The mean frequency of PD-1 inhibitor administration was 3.0 (range, 1-8).

### Safety and efficacy

Complications related to MWA-TACE are shown in **Table 2**. There were no major complications during the hospitalization and follow-up period. Minor complications occurred in 9 (21.4%) patients in the MTP group and 11 (24.4%) patients in the MT group (*P* = 0.738). The complications mainly included new ascites (14.3% vs. 17.8%, *P* = 0.658) and segmental bile duct dilatation (9.5% vs. 6.7%, *P* = 0.924) in the two groups.

**Table 2:** Complications Related to Combination Therapy of MWA and TACE.

Complication	MTP group (n=42)	MT group (n=45)	*P* value
New ascites	6 (14.3)	8 (17.8)	0.658
Liver abscess	1 (2.4)	0 (0.0)	0.483
Segmental bile duct dilatation	4 (9.5)	3 (6.7)	0.924
Hemorrhage	1 (2.4)	2 (4.4)	> 0.999
Pleural effusion	2 (4.8)	2 (4.4)	> 0.999
Hepatic artery-portal fistula	0 (0.0)	3 (6.7)	0.242

Eighty-four AEs related to PD-1 inhibitor occurred in 35 (83.3%) patients in the MTP group (**Table 3**). No treatment-related death or grade 4 AEs occurred. 8 (19.0%) patients required a PD-1 inhibitor interruption or symptomatic treatment due to grade 3 reactive capillary endothelial proliferation (n=4), grade 3 hand-foot syndrome (n=2), grade 3 proteinuria (n=1) and grade 3 platelet count decrease (n=1).

**Table 3:** Adverse Events Related to PD-1 Inhibitor Administration in the MTP Group.

Adverse event	Any grade	Grade 1-2	Grade 3
Reactive capillary endothelial proliferation	15 (35.7)	11 (26.2)	4 (9.5)
Aspartate aminotransferase increase	10 (23.8)	10 (23.8)	0 (0.0)
Alanine aminotransferase increase	10 (23.8)	10 (23.8)	0 (0.0)
Total bilirubin increase	7 (16.7)	7 (16.7)	0 (0.0)
Leukocytopenia	2 (4.8)	2 (4.8)	0 (0.0)
Hypothyroidism	7 (16.7)	7 (16.7)	0 (0.0)
Hyperthyroidism	2 (4.8)	2 (4.8)	0 (0.0)
Proteinuria	4 (9.5)	3 (7.1)	1 (2.4)
Nausea	5 (11.9)	5 (11.9)	0 (0.0)
Fatigue	6 (14.3)	6 (14.3)	0 (0.0)
Diarrhea	5 (11.9)	5 (11.9)	0 (0.0)
Hand-foot syndrome	6 (14.3)	4 (9.5)	2 (4.8)
Platelet count decrease	1 (2.4)	0 (0.0)	1 (2.4)
Decreased appetite	2 (4.8)	2 (4.8)	0 (0.0)
Myocarditis	2 (4.8)	2 (4.8)	0 (0.0)

**Figure 2:**
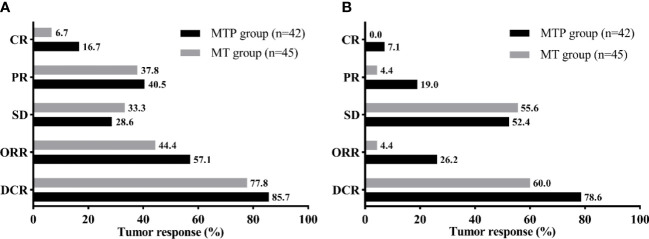
Tumor response of the intrahepatic targeted tumor **(A)** and overall tumor **(B)** for the two groups. MTP, microwave ablation and transarterial chemoembolization combined with PD-1 inhibitor; MT, microwave ablation and transarterial chemoembolization; CR, complete response; PR, partial response; SD, stable disease; ORR, objective response rate; DCR, disease control rate.

All patients had at least one radiologic tumor response assessment during the study (**Figure 2**). 7 (16.7%) patients in the MTP group and 3 (6.7%) patients in the MT group achieved CR of the intrahepatic targeted tumor. The ORR and DCR of intrahepatic targeted tumor in the MTP group were similar to that in the MT group (ORR, 57.1% vs. 44.4%, *P* = 0.236; DCR, 85.7% vs. 77.8%, *P* = 0.340). In addition, MTP group had a higher ORR of overall tumor than MT group (26.2% vs. 4.4%, *P* = 0.011). The DCR of overall tumor was slightly higher in the MTP than that in the MT group, although there was no difference between the two groups (78.5% vs. 60.0%, *P* = 0.061).

### Survival

**Figure 3:**
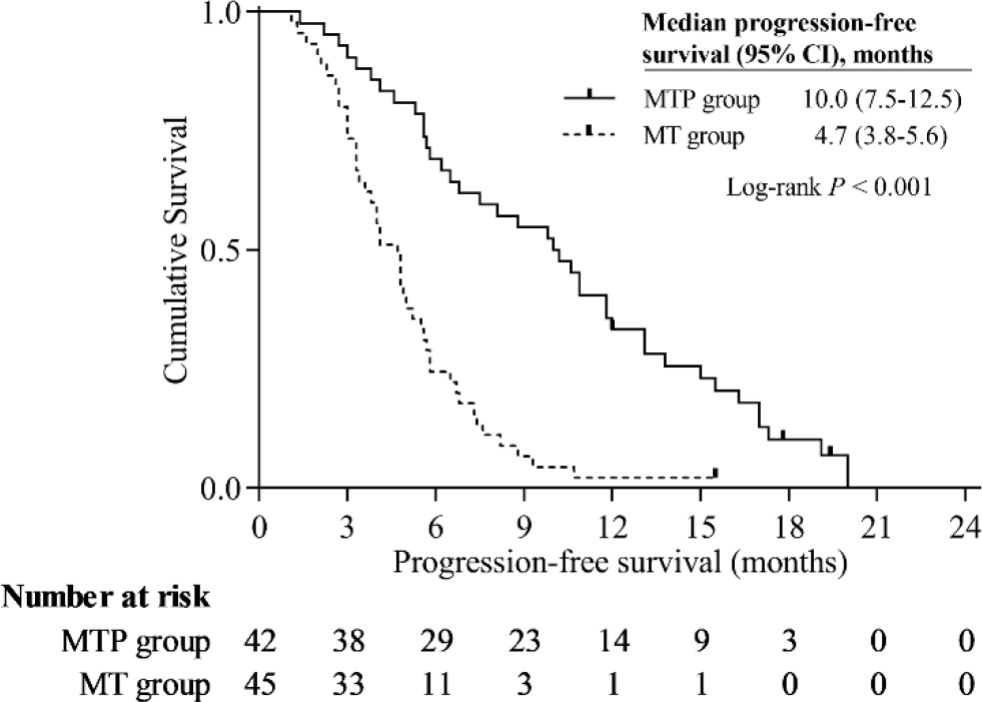
Kaplan-Meier curves of progression-free survival.

**Figure 4:**
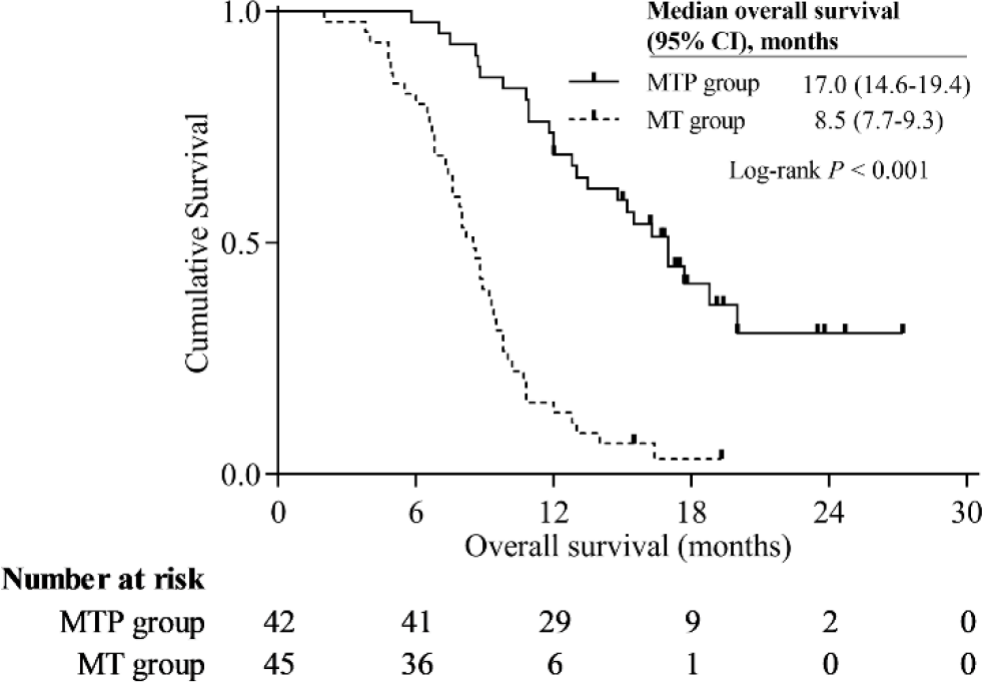
Kaplan-Meier curves of overall survival.

In this study population, the Kaplan-Meier curves indicated that MTP group had significantly better survival benefits than MT group. The median PFS was 10.0 months (95% CI, 7.5-12.5 months) in the MTP group and 4.7 months (95% CI, 3.8-5.6 months) in the MT group (*P* < 0.001) (**Figure 3**). The median OS was 17.0 months (95% CI, 14.6-19.4 months) in the MTP group and 8.5 months (95% CI, 7.7-9.3 months) in the MT group (*P* < 0.001) (**Figure 4**).

Univariate analyses revealed that α-fetoprotein level, treatment method, Child-Pugh class and BCLC stage were significantly associated with OS (**Table 4**). On the basis of these findings, further multivariate analysis indicated that treatment method (MT vs. MTP; hazard ratio [HR] = 5.01, 95% CI, 2.86-8.78, *P* < 0.001) and Child-Pugh class (B vs. A; HR = 2.47, 95% CI, 1.37-4.45, *P* = 0.003) were identified as independent prognostic factors for OS (**Table 5**).

**Table 4:** Univariate Analysis of Prognostic Factors for Overall Survival.

Factor	No. of patients (n=87)	Median overall survival (95% CI), months	*P* value
Etiology			0.424
Hepatitis B infection	72	10.0 (8.8-11.2)	
Other	15	12.0 (10.6-13.4)	
α-fetoprotein level			0.024
< 400 ng/mL	37	13.0 (11.0-15.0)	
≥ 400 ng/mL	50	9.2 (8.4-10.0)	
Treatment method			< 0.001
PD-1 inhibitor with MWA-TACE	42	17.0 (14.6-19.4)	
MWA-TACE	45	8.5 (7.7-9.3)	
Maximal tumor diameter			0.547
< 10 cm	47	12.0 (9.6-14.4)	
≥ 10 cm	40	9.8 (7.5-12.1)	
No. of tumors			0.513
≤ 3	52	10.7 (9.1-12.3)	
> 3	35	10.8 (8.7-12.9)	
Child-Pugh class			0.012
A	70	10.9 (9.7-12.1)	
B	17	8.0 (6.1-9.9)	
ALBI grade			0.198
1	35	12.8 (9.8-15.8)	
2	52	9.4 (8.1-10.7)	
BCLC stage			0.076
B	15	12.0 (6.1-17.9)	
C	72	9.8 (8.3-11.3)	
Extrahepatic spread			0.709
Absent	67	10.7 (9.5-11.9)	
Present	20	10.9 (5.4-16.4)	

CI, confidence interval; MWA-TACE, microwave ablation and transarterial chemoembolization; ALBI, albumin-bilirubin; BCLC, Barcelona clinical liver cancer.

**Table 5:** Multivariate Analysis of Prognostic Factors for Overall Survival.

Factor	HR (95% CI)	*P* Value
α-fetoprotein level		0.149
< 400 ng/mL	1	
≥ 400 ng/mL	1.45 (0.88-2.41)	
Treatment method		< 0.001
PD-1 inhibitor with MWA-TACE	1	
MWA-TACE	5.01 (2.86-8.78)	
Child-Pugh class		0.003
A	1	
B	2.47 (1.37-4.45)	
BCLC stage		0.257
B	1	
C	1.51 (0.74-3.08)	

HR, hazard ratio; CI, confidence interval; MWA-TACE, microwave ablation and transarterial chemoembolization; BCLC, Barcelona clinical liver cancer.

## Discussion

In recent years, TKIs including sorafenib and lenvatinib have been recommended as the first-line therapy for patients with advanced HCC or earlier stage tumor progression (5, 17). Although most of patients can benefit from TKIs, some HCC patients are prone to drug intolerance and discontinuation. This may increase the risk of tumor progression or recurrence. Since atezolizumab combined with bevacizumab resulted in better survival than sorafenib in the IMbrave150 trial, the efficacy and safety of immune-related combination therapy have begun to be widely validated in clinical practice.

A triple combination of MWA, TACE and PD-1 inhibitor was used for TKI-intolerant HCC patients in this study. Compared to other studies of TACE combined with TKIs and/or ICIs, our cohort achieved favorable tumor control and survival benefits in patients with HCC. Marinelli et al. (20) revealed the efficacy of locoregional treatment (TACE or transarterial radioembolization) combined with nivolumab for intermediate and advanced HCC. The median TTP was 4.3 months and 7.4 months in the TACE and transarterial radioembolization, respectively. Two retrospective studies investigated TACE combined with camrelizumab in patients with HCC. Guo et al. (21) reported that twenty HCC patients who underwent a combination of TACE and camrelizumab had an ORR of 56.9% at three months, and the median PFS was 9 months. Compared to the TACE-alone group, it failed to significantly improve the survival benefit in the TACE-camrelizumab group for patients with recurrent HCC. In addition, Zhang et al. (22) reported an ORR of 35.3% in 34 HCC patients who underwent TACE and camrelizumab. The median PFS and OS were 6.1 months and 13.3 months, respectively.

Our study showed that patients who received MWA-TACE combined with PD-1 inhibitor had a relatively longer PFS and OS compared to those patients who received MWA-TACE alone. This may be contributed to the use of PD-1 inhibitor. Previous studies found that massive tumor necrosis increased the release of tumor-associated antigens after MWA/TACE and further activated the immune response against tumor (16, 23). PD-1 inhibitor had a significant synergistic anti-tumor effect because PD-1 can be highly expressed on activated T and B cells (24). Ju et al. (25) indicated that TACE combined with apatinib plus camrelizumab has a promising anti-tumor activity in patients with unresectable HCC. Late combination may provide better OS and PFS when compared to early combination.

The combination of MWA and TACE can achieve effective control of intrahepatic tumor. The DCR of intrahepatic targeted tumor was 85.7% in the MTP group and 77.8% in the MT group, even though 40 (46.0%) patients had tumor over 10 cm in diameter. It was difficult to make massive tumor cell necrosis by only one session. Therefore, repeated MWA and/or TACE “on demand” was performed to control tumor growth in this study. It is widely accepted that repeated local treatment (TACE or ablation) “on demand” is safe and effective (26, 27). In particular, our study performed MWA and TACE simultaneously, which might differ from other studies that conducted TACE and sequential MWA at intervals (28, 29). The potential synergistic effects include the decreased dose of lipiodol and chemotherapeutic agents, and minimal liver function damage with maximum tumor necrosis (30, 31). MWA can destroy some relatively hypovascular HCCs that do not respond well to TACE alone. Meanwhile, the TACE procedure can treat residual tumors after MWA and undetected satellite lesions adjacent to the main large tumor by occlusion of the tumor-feeding artery. Subsequent hepatic arteriography can assure effective treatment for the local tumor.

Our study suggested that MWA-TACE combined with PD-1 inhibitor was safe for the treatment of TKI-intolerant HCC patients. The most common AEs related to PD-1 inhibitor were similar to those reported in previous studies (32, 33). In addition, the MWA and TACE procedures were well tolerated and manageable, suggesting that the combination therapy does not increase the AEs related to MWA-TACE.

There were several limitations in the study. Firstly, this study was a single-center retrospective rather than a randomized study, which may increase the risk of bias. Secondly, some immune-related indicators, such as CD3/CD4 T cells, Treg cells, NK cells, and other immunoregulatory molecules/cells, had not been detected and analyzed in this study. Further studies should be performed to investigate the mechanism of combination therapy in the future. Thirdly, the sample size was relatively small, and subgroup analysis was not performed in detail.

In conclusion, the current study provided an alternative combination therapy for TKI-intolerant HCC patients. Compared to MWA-TACE alone, MWA-TACE combined with PD-1 inhibitor resulted in better anti-tumor effect, longer survival and similar safety profile. These findings need to be confirmed in large sample, prospective randomized controlled trials.

## Data availability statement

The original contributions presented in the study are included in the article/supplementary material. Further inquiries can be directed to the corresponding authors.

## Ethics statement

The studies involving human participants were reviewed and approved by Ethics Committee of Zhongshan Hospital, Fudan University. The patients/participants provided their written informed consent to participate in this study.

## Author contributions

ZY and LL conceived and designed the experiments. QS, XZ, ZZ, WZ, JM, MY and JY collected the data and performed the analysis. QS, XZ and ZZ wrote the paper. JL, LL and ZY reviewed the draft. All authors contributed to the article and approved the submitted version.
